# Supplementary material for: In vitro assembly of the Rous Sarcoma Virus capsid protein into hexamer tubes at physiological temperature
Source: Sci Rep. 2017 Jun 6;7:2913. doi: 10.1038/s41598-017-02060-0 (PMC5460288; doi:10.1038/s41598-017-02060-0)
Supplement: Supplementary file 1 — Supplementary Information [file 41598_2017_2060_MOESM1_ESM.pdf]

Supplementary Information for:

In vitro assembly of the Rous Sarcoma Virus capsid protein into hexamer tubes at physiological temperature.

Soumeya A. Jaballah, Graham D. Bailey, Ambroise Desfosses, Jaekyung Hyun, Alok K. Mitra, & Richard L. Kingston.

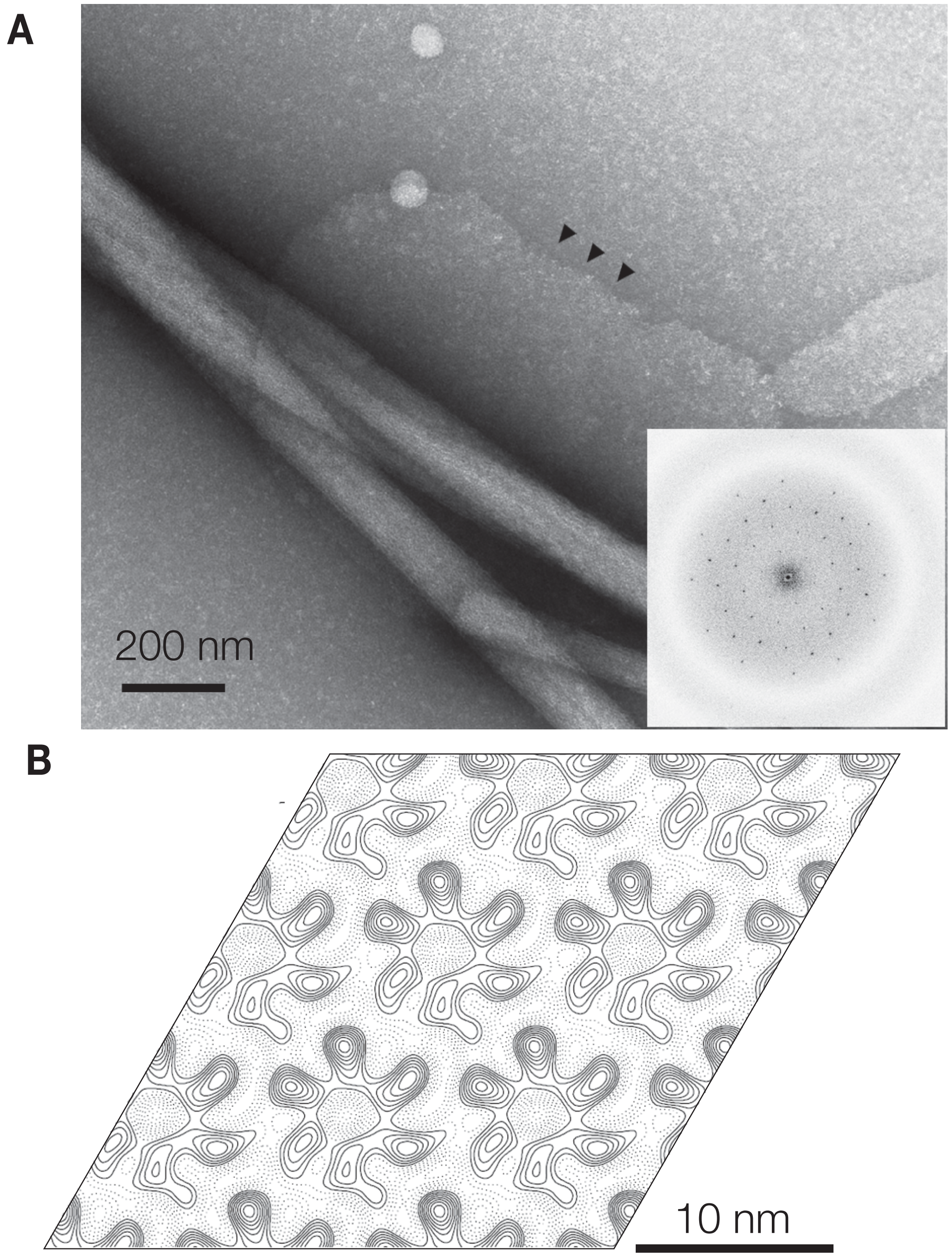

Fig S1 Partially unrolled RSV CA tube visualized by negative-stain EM.

(A) Electron micrograph of a negatively-stained RSV CA tube. A small region that has "unrolled" is indicated with arrowheads. The Fourier transform of this region is shown in the inset. The spots in the diffraction pattern can be indexed on a primitive hexagonal lattice with unit cell dimension a = b = ~96 Å, γ = ~120°.

(B) Unsymmetrized 2D projection map calculated by Fourier synthesis, using crystallographic amplitudes and phases extracted from the Fourier transform in (A).

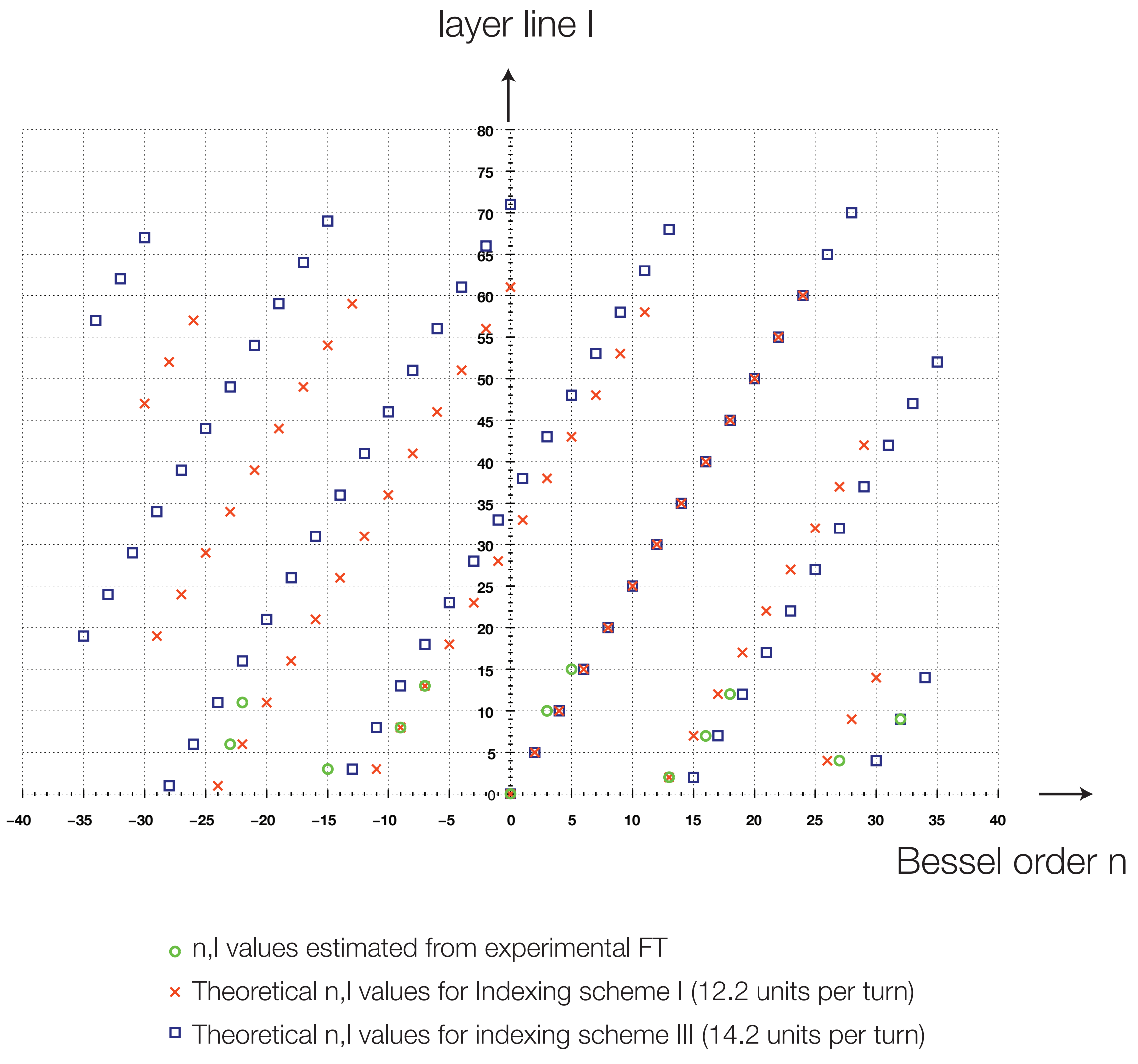

Fig S2 Plot of estimated Bessel order (n) versus layer line number (l) for a RSV CA-SP tube.

Experimental estimates are shown in green. Expected values for indexing scheme I (12.2 units per turn) or indexing scheme III (14.2 units per turn) are shown in red and blue, respectively. Refer Table S2 for details of the indexing schemes.

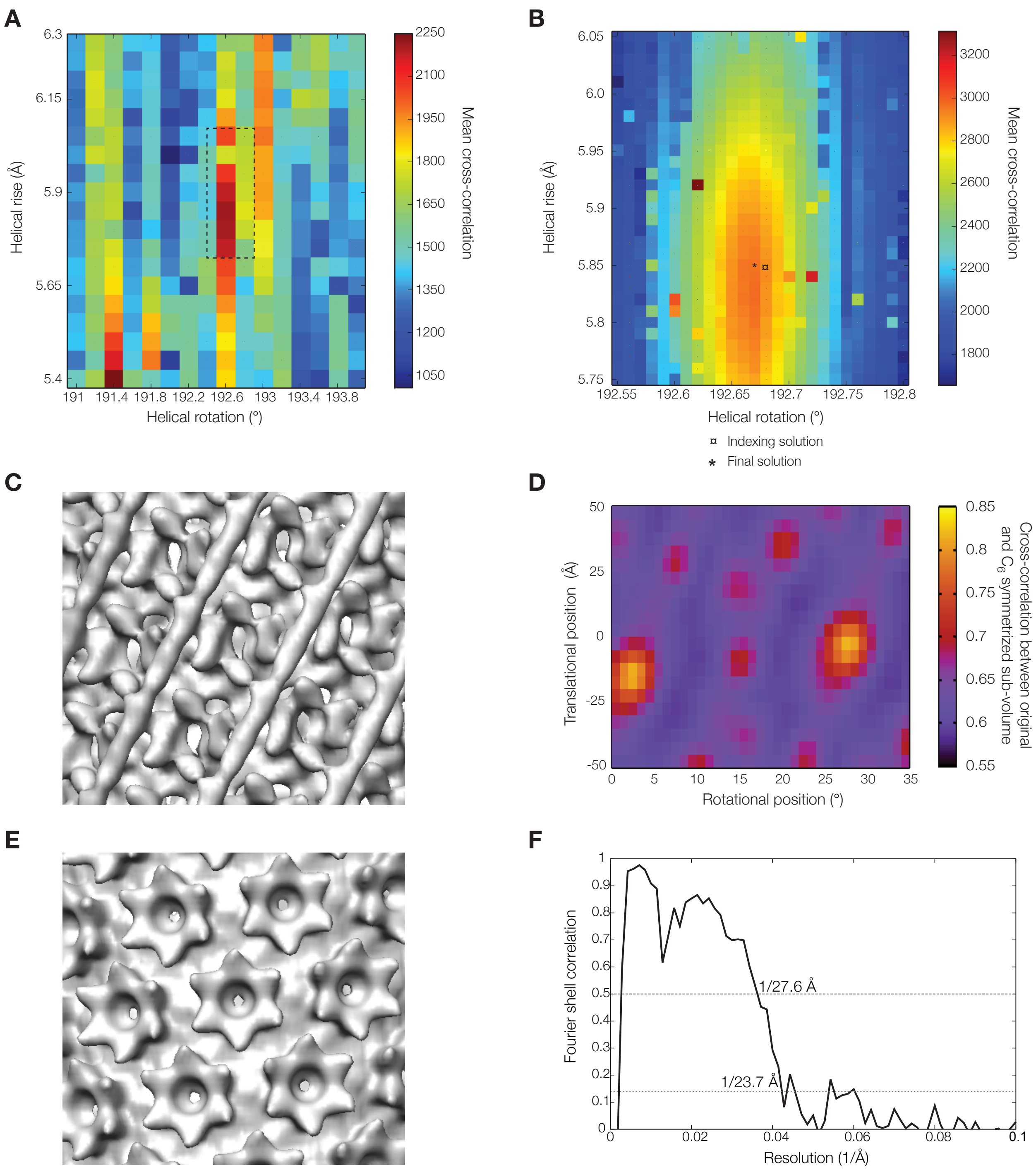

Fig S3 Definition and application of helical symmetry during the 3D reconstruction of a RSV CA-SP tube.

A) The cross-correlation between the projections of the reconstructed volumes and the matching segments as a function of helical rise and rotation.
B) As for part A, but the cross-correlation is explored on a finer grid, corresponding to the boxed region in A. The refined estimates for the helical symmetry parameters (asterisk) are very close to the estimates derived from initial Fourier based indexing (square).
C) The 3D reconstruction before imposition of local C6 symmetry.
D) Plot of the cross-correlation between original and C6-symmetrized sub-volumes as a function of centroid position. The two peaks of equal height are related to each other by the helical symmetry.
E) The 3D reconstruction after imposition of local C6 symmetry.
F) The Fourier Shell Correlation between reconstructions obtained for half of the data set shows a resolution of 27.6Å or 23.7Å using the 0.5 or 0.143 criteria, respectively.

Supplementary Table

Table S1. Protein expression plasmids.

Plasmid Name	Expressed protein	Parental vector	Detail of coding sequence insertion
pGB343[a]	RSV CA	pTXB1 [NEB]	Nde I 1 2 3 235 236 237 SpeI<br>CAT ATG **CCT GTA GTG ... GGC ATA GCC** TGC ATC ACG GGA GAT GCA CTA GTT ...<br>**PRO VAL VAL ... GLY ILE ALA** CYS ILE THR GLY ASP ALA LEU VAL ...<br>RSV CA Mxe GyrA Intein
pGB382[a]	RSV CA+12 (“CA-SP”)	pTXB1 [NEB]	Nde I 1 2 3 235 236 237 238 239 240 241 242 243 244 245 246 247 248 249 SpeI<br>CAT ATG **CCT GTA GTG ... GGC ATA GCC GCG GCC ATG TCG TCT GCT ATC CAG CCC TTA ATT ATG** TGC ATC ACG GGA GAT GCA CTA GTT ...<br>**PRO VAL VAL ... GLY ILE ALA ALA ALA MET SER SER ALA ILE GLN PRO LEU ILE MET** CYS ILE THR GLY ASP ALA LEU VAL ...<br>RSV CA-SP Mxe GyrA Intein

[a] To create expression plasmids encoding RSV CA and RSV CA-SP, fused to the N-terminus of a modified *Mycobacterium xenopi* GyrA intein, the relevant coding sequences (RSV Prague C strain, Genbank Accession Number V01197) were amplified by PCR, and directionally inserted into the vector pTXB1 using the available NdeI and SpeI sites.

Supplementary Table

Table S2. Possible helical symmetries deduced from plotting estimated Bessel order n versus layer line number l, expressed in terms of a 2-strand helix

	Screw Rotation Describing Each Strand				Screw Rotation Relating the 2 Strands*	
	**Rise (Å)**	**Rotation (°)**	**Pitch (Å)**	**Units per turn**	**Rise (Å)**	**Rotation (°)**
**i**	13.6	29.5	166	12.2	6.8	194.75
**II**	12.5	27.3	165	13.2	0	180
**III**	11.7	25.4	166	14.2	5.85	192.68

* For I and III, where no 2-fold rotational symmetry axis exists coincident with the helix axis, these are the parameters of the elementary helix by which the helical lattice could also be described.
